# Choice of DNA extraction method affects stool microbiome recovery and subsequent phenotypic association analyses

**DOI:** 10.1038/s41598-024-54353-w

**Published:** 2024-02-16

**Authors:** Asier Fernández-Pato, Trishla Sinha, Ranko Gacesa, Sergio Andreu-Sánchez, Milla F. Brandao Gois, Jody Gelderloos-Arends, Dianne B. H. Jansen, Marloes Kruk, Martin Jaeger, Leo A. B. Joosten, Mihai G. Netea, Rinse K. Weersma, Cisca Wijmenga, Hermie J. M. Harmsen, Jingyuan Fu, Alexandra Zhernakova, Alexander Kurilshikov

**Affiliations:** 1 Department of Genetics, University of Groningen, University Medical Center Groningen, Groningen, 9713GZ the Netherlands; 2 Department of Gastroenterology and Hepatology, University Medical Center Groningen, Groningen, 9713GZ the Netherlands; 3 Department of Pediatrics, University of Groningen, University Medical Center Groningen, Groningen, 9713GZ the Netherlands; 4 Department of Internal Medicine and Radboud Center for Infectious Diseases, Radboud University Medical Center, Nijmegen, the Netherlands; 5 Department of Medical Genetics, Iuliu Hatieganu University of Medicine and Pharmacy, Cluj-Napoca, Romania; 6 Department of Genomics and Immunoregulation, Life and Medical Sciences Institute, University of Bonn, Bonn, Germany; 7 Medical Microbiology and Infection Prevention, University of Groningen, University Medical Center Groningen, Groningen, 9713GZ the Netherlands

**Keywords:** Gut microbiome, Fecal sample, DNA extraction, Shotgun metagenomics, Microbial genetics, Bioinformatics

## Abstract

The lack of standardization in the methods of DNA extraction from fecal samples represents the major source of experimental variation in the microbiome research field. In this study, we aimed to compare the metagenomic profiles and microbiome–phenotype associations obtained by applying two commercially available DNA extraction kits: the AllPrep DNA/RNA Mini Kit (APK) and the QIAamp Fast DNA Stool Mini Kit (FSK). Using metagenomic sequencing data available from 745 paired fecal samples from two independent population cohorts, Lifelines-DEEP (LLD, n = 292) and the 500 Functional Genomics project (500FG, n = 453), we confirmed significant differences in DNA yield and the recovered microbial communities between protocols, with the APK method resulting in a higher DNA concentration and microbial diversity. Further, we observed a massive difference in bacterial relative abundances at species-level between the APK and the FSK protocols, with > 75% of species differentially abundant between protocols in both cohorts. Specifically, comparison with a standard mock community revealed that the APK method provided higher accuracy in the recovery of microbial relative abundances, with the absence of a bead-beating step in the FSK protocol causing an underrepresentation of gram-positive bacteria. This heterogeneity in the recovered microbial composition led to remarkable differences in the association with anthropometric and lifestyle phenotypes. The results of this study further reinforce that the choice of DNA extraction method impacts the metagenomic profile of human gut microbiota and highlight the importance of harmonizing protocols in microbiome studies.

## Introduction

The human gut harbors diverse microbes that play a fundamental role in the well-being of their host. Numerous studies have identified gut microbiome activities that range from immune functions and protection against pathogens to roles in human metabolism, nutrition and brain function^1–4^. Recently, there has been a focus on gut microbiome research because of its role in influencing the development of numerous diseases^5,6^. The composition of the microbes in the gut has largely been evaluated using fecal samples, which are easy and non-invasive to obtain. However, many parameters of fecal sampling and processing affect the composition of the gut microbiota, including sample collection method, storage (solution composition, storage temperature), homogenization method for DNA extraction (e.g. mechanical or enzymatic lysis), choice of PCR primers (in the case of 16S rRNA analysis) and sequencing method^7,8^.

Isolation and purification of DNA from fecal samples is a crucial initial step to ensure high yield and quality of isolated nucleic acids. Over the past decades, several DNA extraction kits have been developed that are now commercially available, with the goal of enabling rapid extraction of large numbers of fecal samples. Available kits differ in several steps of the extraction process, including the cell lysis procedure and DNA isolation and purification methods. Earlier research showed that different DNA extraction kits yield different results in terms of the amount and quality of DNA extracted and the bacterial community composition^9,10^, with the inclusion of a mechanical lysis step demonstrated to have a positive effect on the recovery of DNA from gram-positive bacteria^10–13^. This heterogeneity between methods causes substantial technical variation among cohorts, an issue that represents a major challenge in multicenter microbiome studies, and it may be partially responsible for the low replication rate in microbiome research^9,14,15^. In addition, the effects of DNA extraction methods on the microbial composition determined by shotgun metagenomic sequencing have not been extensively explored in large cohorts. In this study, we investigated the impact of two fecal DNA extraction methods on the yield and quality of isolated DNA and microbiome composition and diversity. We also evaluated the accuracy of each method using a mock community of known composition and the implications of these effects for microbiome–phenotype associations. For this purpose, two commercially available DNA extraction kits were applied to fecal samples from two independent cohorts of 292 and 453 participants (745 pairs of samples in total) in which other sources of heterogeneity were reduced as much as possible, followed by shotgun metagenomic sequencing.

## Materials and methods

### Study population

**Figure 1:**
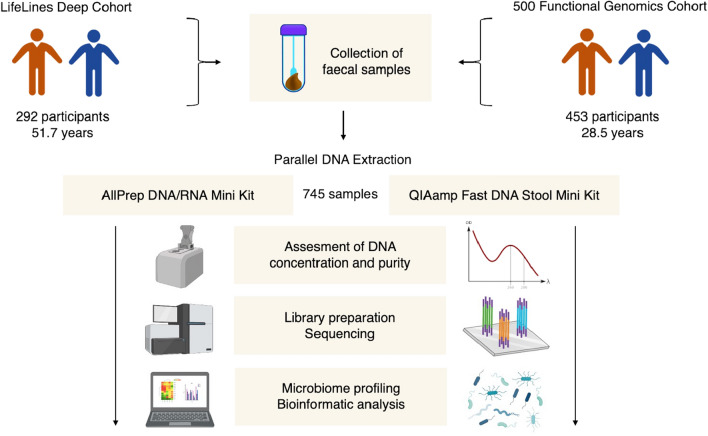
Experimental study design. Fecal samples collected from participants of two different cohorts were included in the present study. In total, 745 samples were isolated using two different DNA extraction kits: AllPrep DNA/RNA Mini Kit and QIAamp Fast DNA Stool Mini Kit (QIAGEN, Germany). After measuring DNA concentration and quality, extracted samples underwent library preparation and sequencing using the same technology (Illumina Hiseq 2000) at two different sequencing facilities. Finally, microbiome profiling and statistical analysis of sequencing data was performed.

In this study we included available metagenomics sequencing data from two large Dutch cohorts: Lifelines-DEEP follow-up (LLD, a subcohort of the Lifelines biobank, Groningen, the Netherlands) and 500 Functional Genomics (500FG, Nijmegen, the Netherlands)^16,17^. LLD comprises 338 individuals (55.6% female, 44.4% male, mean age: 51.7 years) sampled in 2017, 4–5 years after the original Lifelines-DEEP study^18^. The 500FG cohort consists of 534 healthy individuals (44.4% male, 55.6% female, mean age: 28.5 years), with stool samples taken between July 2013 and December 2014 available for 471 participants. Both cohorts combine deep phenotypic characterization with extensive collection of biological samples, including stool samples for metagenomic analysis. For the present study, we selected 292 fecal samples from LLD and 453 samples from 500FG that had all been isolated using two different DNA extraction protocols (Fig. 1).

### Ethics declarations and consent to participate

The Lifelines-DEEP study was approved by the ethics committee of the University Medical Center Groningen, document number METC UMCG LLDEEP: M12.113965. All participants signed an informed consent form prior to study enrollment. The 500FG study was approved by the Ethical Committee of Radboud University Nijmegen (NL42561.091.12, 2012/550). Inclusion of volunteers and experiments were conducted according to the principles expressed in the Declaration of Helsinki. All volunteers gave written informed consent before any material was taken.

### Sample collection and DNA isolation

All stool samples included in this study were collected by participants at home and frozen within 15 min after production. Frozen samples were then collected by qualified personnel, transported on dry ice, and stored at − 80 °C until DNA extraction. We compared two commercially available DNA extraction methods used in the human microbiome research field: the AllPrep DNA/RNA Mini Kit (QIAGEN, Germany) and the QIAamp Fast DNA Stool Mini Kit (QIAGEN, Germany), hereafter referred to as APK and FSK, respectively. Samples from both cohorts were extracted shortly after sample collection using the exact same procedure, except for the 500FG samples processed with FSK, which were stored frozen for 4 years and the isolation was performed in a different laboratory. For both extraction kits, DNA extraction was performed according to the manufacturer’s instructions with the following deviations: (1) we performed an enzymatic lysis (with TE buffer containing lysozyme and proteinase K) and a bead-beating step in the APK-based protocol and (2) the FSK protocol was automated using the QIAcube, increasing the temperature for the cell lysis step from 70 to 95 °C (See Supplementary Material for detailed methods).

### Assessment of DNA yield and purity

The concentration of DNA extracted from LLD samples was measured spectrophotometrically using a NanoDrop ND-1000 (Thermo Fisher Scientific, United States). The DNA concentrations of samples from the 500FG cohort were assessed differently according to the DNA extraction method used. Samples extracted with the FSK protocol were evaluated with the NanoDrop ND-1000. Samples processed with the APK method were analyzed via Quant-iT PicoGreen dsDNA Assay (Thermo Fisher Scientific). In both cohorts, DNA purity was assessed with the NanoDrop ND-1000 and calculated as the 260/280 absorbance ratio.

### Library preparation, sequencing and microbiome profiling

Following DNA extraction, differentially processed samples from both cohorts were sent to two alternative sequencing facilities (APK: Broad Institute of Harvard and MIT, United States; FSK: Novogene, China) to perform library preparation (FSK: NEBNext® DNA Library Prep Kit (New England BioLabs, United States); APK: Nextera® XT DNA Library Preparation Kit (Illumina, United States)) and whole-genome shotgun sequencing on the Illumina HiSeq 2000 platform. Following the filtration of low-quality reads at the sequencing facilities, we removed raw metagenomic reads that mapped against the reference human genome (GRCh37) or aligned to Illumina adapters using KneadData (v.0.7.4). The remaining reads were used as input to determine the taxonomic profiles of the samples using the Biobakery4 tool MetaPhlAn4 (using the January 2021 CHOCOPhlAn database)^19^.

### Mock community

The accuracy and bias of each DNA extraction method was assessed using a commercially available mock community (ZymoBIOMICS Catalog #D6300). This mixed microbial community comprises eight bacteria (five gram-positive and three gram-negative) and two yeasts, representing a diverse range of microorganisms with varying levels of resistance to cell-wall lysis. Three replicates were extracted using the APK protocol and two replicates using the FSK protocol. A third mock sample was processed using the FSK protocol with an additional bead-beating step (Kiel, Germany) to assess the independent impact of this physical disruption step. We measured the recovered DNA concentration with the Qubit 2.0 Fluorometer (Thermo Fisher Scientific, United States) and applied the exact same procedure described above for library preparation (NEBNext® DNA Library Prep Kit), whole-genome shotgun sequencing (Novogene, China) and subsequent bioinformatic analysis.

### Statistical analysis

Statistical differences in read depth, DNA concentration and purity measures were assessed using the Wilcoxon signed-rank test. DNA yields obtained from each extraction method were compared to a predicted DNA recovery that assumes that an average stool sample contains 10^11^ bacterial cells per gram, amounting to a total 10^10^ bacterial cells in our stool samples (an average of 100 mg). A total DNA yield of 0.01 mg was calculated assuming each bacterial cell consists of 1 fg DNA. The same statistical test was applied to determine microbial richness and diversity differences estimated with R package mia (v.1.9.2)^20^ (based on the vegan package)^21^. We then estimated the Pearson’s correlation of (1) alpha diversity and read depth and (2) alpha diversity between paired samples extracted with the different protocols. Differences in microbial community composition were analyzed and visualized via principal coordinate analysis (PCoA) based on Aitchison and Bray–Curtis distances. The contribution of DNA extraction protocol and anthropometric variables to inter-individual microbiome community variation on species-level was determined by a PERMANOVA analysis using the adonis function. A Mantel test was applied to evaluate the correlation of the distance matrices of samples extracted with each DNA extraction method. Differences in the relative abundances of microbial taxa between paired samples were analyzed at phylum and species-level using a paired t-test on centered log-ratio (CLR)-transformed counts. We also assessed Pearson’s correlations of log-transformed microbial relative abundances between APK and FSK. Next, we determined the associations of the available metadata with microbial diversity measures (Pearson’s correlation and PERMANOVA analysis for alpha and beta diversity, respectively) and CLR-transformed relative abundances at species-level (linear regression). Association analysis between the relative abundances of bacterial species and host metadata was further performed by integrating both cohorts and DNA extraction protocols using linear mixed models (lmer function): CLR_transformed_species_abundance ~ phenotype + (1 | Cohort).

Only species present at a prevalence > 10% were considered in the association analyses. Unless otherwise specified, results were considered significant at a false discovery rate (FDR)-corrected p-value < 0.05. All analyses were done using the R programming language (v.4.2.2) (https://www.R-project.org/).

## Results

### Assessment of differences in read depth, DNA concentration and purity

We compared the metagenomic sequencing data from paired fecal samples in the LLD and 500FG cohorts, where DNA from the same sample was isolated using two different methods—APK and FSK. Since sequencing depth can critically affect the microbiome profiling, we first evaluated the read depth values in differentially extracted samples from each cohort. Metagenomic sequencing yielded a significantly different mean read count in the 500FG cohort. After removal of adapter sequences and human reads, 500FG samples processed with the FSK protocol had a significantly higher read depth compared to samples extracted with APK (mean ± SD (million): APK: 11.59 ± 3.64, FSK: 25.19 ± 5.51, paired Wilcoxon test, p < 0.001, Supplementary Fig. 1A). By contrast, we saw no significant differences between protocols in the LLD cohort (mean ± SD (million): APK: 13.33 ± 4.75, FSK: 12.91 ± 2.40, paired Wilcoxon test, p = 0.332, Supplementary Fig. 1A).

Next, we compared DNA concentration and purity between paired samples. LLD samples showed significantly higher DNA concentrations when processed with the APK protocol in comparison to the FSK protocol (mean ± SD: APK: 205.20 ± 73.33 ng/µl, FSK: 64.91 ± 35.70 ng/µl, paired Wilcoxon test, p < 0.001, Supplementary Fig. 1B). Although we saw similar results for the 500FG cohort (mean ± SD: APK: 179.26 ± 87.80 ng/µl, FSK: 106.64 ± 54.92 ng/µl, paired Wilcoxon test, p < 0.001, Supplementary Fig. 1B), the use of different measurement methods for DNA concentration (NanoDrop ND-1000 for APK and Quant-iT PicoGreen dsDNA Assay for FSK) prevents direct comparison of these results. Despite the differences between the DNA extraction protocols, both protocols achieved DNA recoveries that closely matched the expected theoretical value of 0.01 mg (assuming 1 fg DNA per bacterial cell) in the 500FG cohort (mean ± SD: APK: 0.018 ± 0.009 ng/µl, FSK: 0.011 ± 0.005 ng/µl, paired Wilcoxon test, p < 0.001). In the LLD cohort, the FSK method demonstrated a slightly lower DNA yield (mean ± SD: APK: 0.021 ± 0.007 ng/µl, FSK: 0.006 ± 0.004 ng/µl, paired Wilcoxon test, p < 0.001).

Regarding DNA quality, the 260/280 absorbance values were significantly different in LLD, where APK-extracted samples showed a ratio closer to 1.8 (considered pure DNA), while FSK samples displayed a lower DNA purity and a higher standard deviation (mean ± SD: APK: 1.89 ± 0.05, FSK: 1.99 ± 0.13, paired Wilcoxon test, p < 0.001, Supplementary Fig. 1C). Conversely, no significant differences in mean DNA quality were found for 500FG samples (mean ± SD: protocol APK: 1.90 ± 0.06, protocol FSK: 1.91 ± 0.11, paired Wilcoxon test, p = 0.113, Supplementary Fig. 1C). Supplementary Table 1 provides detailed information on read number and DNA concentration, yield and purity.

### DNA extraction method significantly affects gut microbial diversity

**Figure 2:**
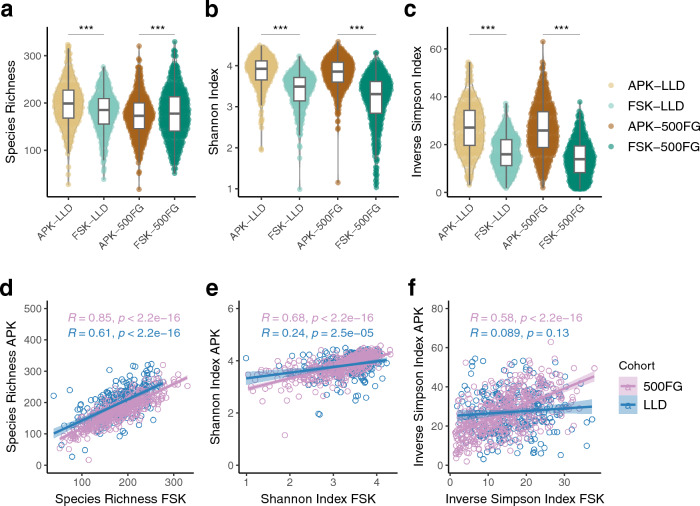
Alpha diversity analysis. Comparison of species richness (**a**), Shannon (**b**) and Inverse Simpson (**c**) diversity values between samples extracted with APK (brown) and FSK (green) from each cohort (LLD: lighter colors, 500FG: darker colors). Each dot represents one sample. Boxplots represent the median (middle horizontal line), the interquartile range (IQR) (boundaries of the boxes) and values within 1.5 times the IQR (whiskers). Correlation plots of species richness (**d**), Shannon (**e**) and Inverse Simpson (**f**) diversity indices between differentially extracted samples from each cohort (LLD: blue, 500FG: violet). Each data point represents one sample. The coefficients and p-values from the Pearson’s correlation are shown accordingly (***p < 0.001, rank-based Wilcoxon test).

Species richness analysis highlighted significant differences between the APK and FSK protocols in the number of species observed in LLD (mean ± SD (species/sample): APK: 197.78 ± 47.36, FSK 181.64 ± 42.38, paired Wilcoxon test, p < 0.001, Fig. 2a), with APK yielding a higher number of species, and in 500FG (APK: 172.77 ± 42.92, FSK 177.80 ± 51.81, paired Wilcoxon test, p < 0.001, Fig. 2a), where FSK showed a larger number of species. However, both the Shannon and Inverse Simpson diversity indices showed significantly higher diversities in APK-extracted samples in both LLD and 500FG (paired Wilcoxon test, p < 0.001, Fig. 2b,c). All diversity values are summarized in Supplementary Table 2. To disentangle the possible influence of differences in read depth on observed diversity, we analyzed the correlation between the alpha diversity indices and read depth, but this did not yield significant results (Supplementary Fig. 2). Notably, species richness was significantly and positively associated with read depth in 500FG samples, regardless of the protocol used. Despite these differences, we found a significant positive correlation of species richness and alpha diversity values between paired samples extracted with the APK and FSK protocols in each cohort (p < 0.001, Fig. 2d–f), with the only exception being the Inverse Simpson diversity values, which did not reach statistical significance in the LLD samples (paired Wilcoxon test, p = 0.13, Fig. 2f).

### Microbial community composition reflects differences driven by DNA isolation protocol

**Figure 3:**
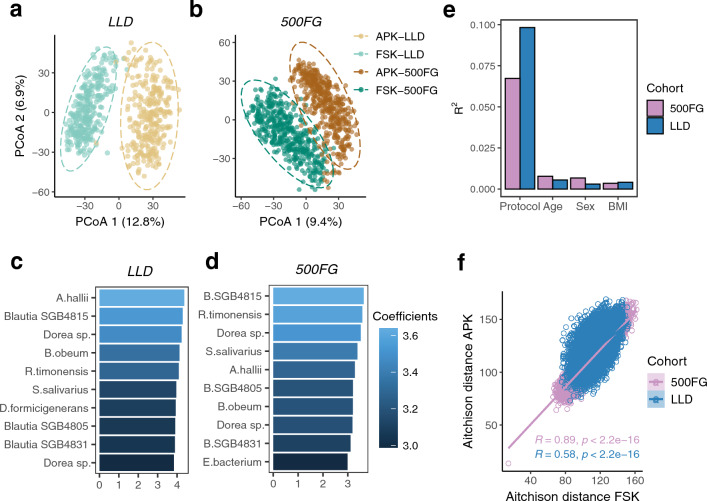
Beta diversity analysis. PCoA plot based on Aitchison distances for (**a**) LLD and (**b**) 500FG samples extracted with each DNA isolation method (APK: brown, FSK: green). Each datapoint represents the microbial community composition of one sample. The ellipses illustrate the standard deviation of samples belonging to each group. (**c**,**d**) Model coefficients estimated by PERMANOVA analysis for species that exhibit the largest differences between samples extracted with the APK and FSK protocol in each cohort. The top 10 species are displayed in order according to the coefficient value (positive: increased in APK). (**e**) Individual effect sizes of DNA extraction protocol, age, sex and BMI on microbiome community variation (LLD: blue, 500FG: violet, PERMANOVA on Aitchison distance). (**f**) Correlation plot of Aitchison distances between differentially extracted samples from each cohort (LLD: blue, 500FG: violet). Each data point represents one sample. The coefficients and p-values from the Mantel test based on Pearson’s correlation are shown accordingly.

We assessed the differences in observed microbial communities between samples extracted with each protocol. PCoA plots based on Aitchison distances showed clear clustering according to the DNA extraction method (Fig. 3a,b), with unpaired samples collected from different participants showing smaller distances than paired samples from the same individuals. PERMANOVA analysis confirmed the significant effect of DNA isolation protocol on microbial community composition (species-level Aitchison distance, LLD: R^2^ = 9.82%, 500FG: R^2^ = 6.73%, p < 0.001, permutations = 1000). The dissimilarities between both groups were mainly driven by bacteria with different Gram-staining-based classifications, with gram-positive bacteria being enriched in samples extracted with the APK protocol (Fig. 3c,d). Although we observed significant effects of other phenotypes including sex, age and body mass index (BMI) in shaping microbial community structure, their effect sizes were considerably smaller (Fig. 3e, Supplementary Table 3).

Additionally, we evaluated the correlation of the distance matrices of samples extracted with the APK and FSK methods separately for each cohort. This analysis yielded a significant correlation between the distances of differentially extracted LLD and 500FG samples, indicative of a limited sensitivity of inter-individual distances to the heterogeneity introduced by the DNA extraction method (Mantel test based on Pearson’s correlation, LLD: r_mantel_ = 0.58, p < 0.001, 500FG: r_mantel_ = 0.89, p < 0.001, permutations = 1000, Fig. 3f). These results were then further validated using the Bray–Curtis dissimilarity index (Supplementary Fig. 3, Supplementary Table 3), confirming that the DNA extraction procedure was a major contributor to microbial community composition changes.

### DNA extraction method has a large effect on observed microbial relative abundances

**Figure 4:**
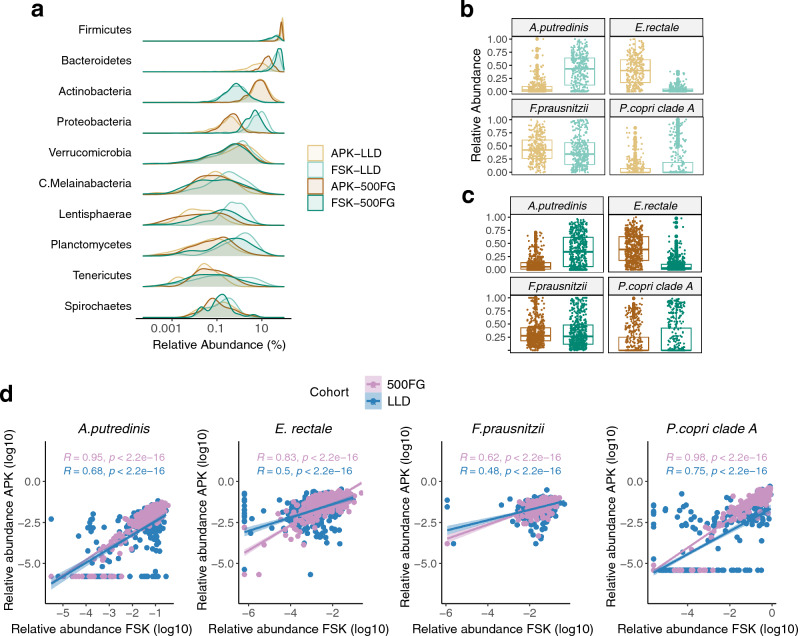
Differential microbial abundance analysis. (**a**) Relative abundances for the microbial phyla visualized as a density plot over a log-scaled axis. Phyla are ordered from top to bottom by decreasing relative abundance. (**b**,**c**) Strip chart showing the relative abundances for the top four most abundant microbial species in both cohorts. Each dot represents a sample. Boxplots represent the median (middle horizontal line), the IQR (boundaries of the boxes) and values within 1.5 times the IQR (whiskers). Relative abundances are colored according to DNA extraction protocol (APK: brown, FSK: green) and cohort (LLD: lighter colors, 500FG: darker colors). (**d**) Correlation plot of log-transformed relative abundances [for species defined in (**c**)] between differentially extracted samples from each cohort (LLD: blue, 500FG: violet). Each data point represents one sample. The coefficients and p-values from the Pearson’s correlation are displayed.

We evaluated the relative abundance of microbial taxa at several taxonomic levels. Phylum-level analysis pinpointed a significant difference between the APK and FSK protocols in the relative abundances of most microbial phyla identified, with the only exceptions being Melainabacteria, Planctomycetes and Tenericutes in 500FG and Verrucomicrobia in the LLD cohort (paired t-test, FDR-adjusted p < 0.05). For instance, we observed higher relative abundances of Firmicutes and Actinobacteria in samples extracted with APK, whereas samples extracted with FSK showed increased relative abundances of Bacteroidetes and Proteobacteria phyla, among others. These observations also showed high consistency between the LLD and 500FG cohorts (Fig. 4a, Supplementary Table 4). Additionally, we found that the Bacteroides to Firmicutes ratio varied significantly between DNA extraction protocols in both cohorts (Wilcoxon signed-rank test, p < 0.001), with APK showing a higher ratio (mean ratio, LLD APK: 9.79, LLD FSK: 0.97; 500FG APK: 3.74, 500FG FSK: 0.67). Analysis at higher taxonomic resolution revealed a higher number of both genera and species detected in > 5% of participants for the APK protocol in LLD (APK: 331 genera, 596 species; FSK: 301 genera, 529 species), while 500FG samples displayed an opposite trend (APK: 286 genera, 508 species; FSK: 297 genera, 521 species). Similar to what we observed at phylum-level, we found that species relative abundances were markedly different between the DNA extraction methods, with 464 and 494 species being differentially abundant in LLD and 500FG, respectively (paired t-test, FDR-adjusted p < 0.05, Supplementary Table 5). Nonetheless, most species showed a positive and significant correlation between both extraction protocols (Supplementary Table 6). When restricting the analysis to the most abundant species in both cohorts, samples extracted with the FSK protocol displayed an increased presence of *Alistipes putredinis* and *Prevotella copri*, whereas *Eubacterium rectale* and *Faecalibacterium prausnitzii* were more abundant in samples extracted with the APK protocol (paired t-test, FDR-adjusted p < 0.05, Fig. 4b,c). Again, however, the relative abundances of these species were positively correlated for both DNA extraction methods (p < 0.001, Fig. 4d). Relative abundances of all microbial species identified are provided in Supplementary Table 7.

To further characterize the differences between protocols, we explored the taxa prevalence profiles separately in each cohort using several relative abundance thresholds. This revealed a different pattern of prevalent taxa between APK and FSK, with a higher number of microbial species reaching 90% prevalence in samples extracted with APK (LLD APK: 32 species, FSK: 25 species; 500FG APK: 28 species, FSK: 25 species). The alteration profile of taxa presence according to relative abundance thresholds was also more alike between samples processed with the same kit, with FSK samples consistently displaying a smaller number of species present at higher relative abundance values (Supplementary Fig. 4).

### The APK protocol better reflects the composition of the mock community

To evaluate the accuracy and biases of the two DNA extraction methods, three replicates of a commercially available mock community were extracted using the APK protocol and two using the FSK protocol, with an additional mock sample processed with FSK that included a bead-beating step in the DNA extraction procedure (Supplementary Table 8). The theoretical composition and observed relative abundances of species for each sample are presented in Supplementary Table 9. In accordance with our previous findings, mock community samples extracted with the APK protocol exhibited increased DNA concentration and alpha diversity, which closely resembled the expected diversity of the commercial microbial community (Supplementary Fig. 5A,B). The APK approach best mimicked the theoretical microbial community in comparison to the FSK method (mean species-level Bray–Curtis distance, APK: 0.173, FSK: 0.466), although neither accurately replicated its known composition. Interestingly, the FSK sample with an additional bead-beating step displayed higher Bray–Curtis distances to samples extracted with APK than to non-bead-beaten samples processed with FSK (mean species-level Bray–Curtis distance, vs APK: 0.354, vs non-bead-beaten FSK: 0.305), but this trend was reversed after CLR-transformation, with a smaller distance observed between bead-beaten samples (Supplementary Fig. 5C). Our ability to perform statistical analysis was hindered by the limited number of replicates per DNA extraction protocol. However, we observed an overabundance of Proteobacteria and a deficiency of Firmicutes in samples processed with the FSK method, supporting the findings about this protocol in the LLD and 500FG cohorts. At the species level, we observed an overrepresentation of *Salmonella enterica*, *Pseudomonas aeruginosa*, *Lactobacillus fermentum* and *Escherichia coli* and an underrepresentation of *Staphylococcus aureus*, *Listeria monocytogenes* and *Enterococcus faecalis* in these samples. The differences were again reduced when including a bead-beating step in the FSK protocol (Supplementary Fig. 5D,E).

### DNA extraction procedure alters phenotypic associations with microbial features

Further, we analyzed whether the DNA extraction protocol affects the observed phenotypic associations with microbial diversity measures and taxa relative abundances. For this, we assessed anthropometric phenotypes (age, sex and BMI) known to be linked to characteristic gut microbiome features and environmental exposures including dietary information and lifestyle habits. Interestingly, we found at nominal significance (p < 0.05) that all associations with alpha (Shannon Index) and beta diversity were conserved between both protocols in the 500FG cohort, except for the consumption of fruit and vegetables, which was only associated to alpha diversity in APK samples (Supplementary Tables 9, 10). Conversely, we observed larger differences in the LLD cohort. The significant correlation of age with alpha diversity found for the APK protocol was not replicated in FSK-extracted samples. Both alpha and beta diversity were exclusively associated with cholesterol level in samples processed with the APK protocol, whereas only beta diversity was significantly associated with BMI in FSK-extracted samples. Despite not reaching statistical significance, some phenotypes showed opposite directions in the association (alpha diversity with sport, social contact, consumption of vegetables, alcohol or sugary products) (Supplementary Tables 10,11).

Remarkably, we observed that the correlations with bacterial species relative abundances were also influenced by the DNA extraction method. In the LLD cohort, the abundance of five bacterial species belonging to order Clostridiales were significantly associated with host phenotypes (including age, BMI and glucose level) in samples extracted with the FSK protocol, but none of these associations were replicated in APK samples, which did not show any significant associations. In 500FG, we found 90 and 100 significant associations between host phenotypes and bacterial species relative abundances in samples extracted with the APK and FSK methods, respectively. However, only 42 were replicated by both DNA extraction protocols (Supplementary Tables 12, 13).

We next explored how the integration of bacterial relative abundance data generated using different DNA extraction procedures could affect the association with host phenotypes. To ensure comparability, only phenotypic information that was encoded in a consistent format across both cohorts was included in the analysis (host age, sex, BMI and smoking status). By combining all samples extracted with the APK protocol from the LLD and 500FG cohorts, we found the relative abundance of 182 bacterial species to be significantly associated with host phenotypes. Similarly, the combination of all available samples extracted with the FSK procedure resulted in 273 significant associations with bacterial relative abundances (115 shared with APK) (Supplementary Fig. 6A, Supplementary Table 14). After the integration of samples extracted with different methodological procedures, only 62.1% (113) (when combining LLD-APK and 500FG-FSK samples) and 75.3% (137) (when combining LLD-FSK and 500FG-APK samples) of the associations preserved both the statistical significance and the direction of the association in comparison to the results of samples extracted with the APK protocol. Similarly, when comparing integrated results to the association profile of FSK-extracted samples, we identified a consistency rate of 59.3% (162) for associations in LLD-APK and 500FG-FSK samples and of 53.5% (146) for associations in LLD-FSK and 500FG-APK samples (Supplementary Fig. 6B, Supplementary Table 15). These results underscore a significant discrepancy in the association outcomes, indicative of the methodological impact on the host-microbiome association profiles.

## Discussion

In this study, we compared two commercially available DNA extraction methods used in microbiome research: the AllPrep DNA/RNA Mini Kit (APK) and the QIAamp Fast DNA Stool Mini Kit (FSK). By leveraging the shotgun metagenomic sequencing data available in two large cohorts (745 paired samples), we assessed differences in DNA yield and quality and taxonomic composition while trying to limit the effects of other sources of heterogeneity. We also evaluated the accuracy of each method and whether associations with several phenotypes were affected by the DNA extraction protocol.

The APK extraction method yielded a higher DNA concentration than the FSK method. Although only a few studies have assessed these two protocols, this difference can be partially explained by the inclusion of a bead-beating step in the APK procedure. This mechanical disruption has been shown to improve DNA extraction efficiency, independent of the commercial kit used^9,11,22^. Specifically, the bead-beating step has been pinpointed as the main determinant of the efficiency of lysis for gram-positive bacteria^9,11–13,23^, which are characterized by the thick peptidoglycan layer that surrounds their plasma membrane, with DNA extraction efficiency observed to vary with the time and strength of the bead-beating^23^. Although previous studies have described that use of a heating step in combination with the enzymatic lysis used in FSK can also favor bacterial cell lysis by denature the membrane proteins^24^, our findings suggest inefficient bacterial DNA recovery with this procedure. While automatization of the FSK protocol could have also contributed to DNA yield dissimilarities, prior research has not found significant differences in nucleic acid concentration and quality between automated and manual methods^12^. Additionally, our results show that the increased DNA concentration in samples extracted with APK resulted in higher microbial diversity, achieving a higher accuracy in replicating the theoretical microbial diversity according to our standard community study. These findings further support earlier evidence that suggests a higher DNA yield and species diversity in bead-beaten samples^9,11,12^. These results, however, could only be validated in the LLD cohort due to technical variability in the DNA concentration measurements in 500FG. In addition, we did not find a significant correlation between alpha diversity indices and read depth, discarding a potential impact of read depth differences on observed diversity values.

Our analysis revealed that DNA extraction method is a major driver of community differences and that its explanatory power (LLD: 9.82%, 500FG: 6.73%) was considerably higher than that of other anthropometric variables including sex (LLD: 0.30%, 500FG: 0.67%), age (LLD: 0.55%, 500FG: 0.77%) and BMI (LLD: 0.41%, 500FG: 0.35%). In contrast with earlier findings, paired samples isolated with different protocols showed lower similarity than unpaired samples extracted with the same protocol, as seen in the clustering on our PCoA. This discrepancy may be partially explained by the limited number of human participants included in the earlier comparative analyses (which ranged from 1 to 18 participants) compared to the 745 paired stool samples included in our analysis^9,12,25–28^. These low numbers of participants substantially limited the ability to compare inter-subject and technical variation, thus hampering the extrapolation of conclusions to large-scale analyses. Nevertheless, some studies have already reported results where the technical variability could be at least comparable to inter-individual variation at taxonomic^29^ and functional level^9^. In addition, we found that the inter-individual distances had a limited sensitivity to the heterogeneity introduced by the DNA extraction method, as distance matrices of differentially extracted samples were positively correlated. Our comparative analysis with a known community of microbes with varying resistance to cell-wall lysis further revealed that none of the methods we used accurately matched the theoretical microbial composition. However, in agreement with Tourlousse et al.^23^, the protocol with a bead-beating step (APK) exhibited the highest accuracy, whereas the FSK approach showed the largest divergence from the known composition. Remarkably, including the physical disruption step in the FSK procedure minimized the differences from the standard community abundances.

Several studies have previously described differences in taxonomic composition associated with the DNA extraction protocol. However, they have mainly been restricted to genus-level taxonomic resolution due to the limitations of 16S rRNA gene amplicon–based analysis, the predominant method used in existing literature^8,10,12,30,31^. Evidence for substantial species-level heterogeneity in the human microbiome, and even for the presence of strain-specific phenotypes and functional profiles, highlights the need to gain deeper insights into lower taxonomic levels^32^. Therefore, in the present study, we report a massive alteration of species-level relative abundances in stool samples processed with APK and FSK, with a large proportion of the identified species being differentially abundant according to the DNA extraction protocol used. Interestingly, due to the compositional nature of microbiome data, protocol-dependent efficiencies in the disruption of the cell walls of gram-positive bacteria resulted in a considerable fraction of the differentially abundant species being gram-negative bacteria. Nonetheless, our findings broadly support previous work showing an increased relative abundance of gram-positive bacteria after the inclusion of a mechanical lysis step, with an underrepresentation of Firmicutes phyla by the FSK method. Remarkably, we also found that methodological differences in DNA isolation impacted phenotypic associations with alpha and beta diversity measures. We found that DNA extraction with the FSK protocol led to a loss of significance in the association of microbial diversity with several anthropometric and lifestyle factors, including age (both cohorts), cholesterol level (LLD cohort) and fruit consumption (500FG cohort). Similarly, the use of this DNA extraction method hindered identification of the significant associations between microbial composition and cholesterol level (LLD cohort) and with consumption of vegetables and caffeinated drinks (500FG cohort) that were present in samples processed with the APK procedure. Additionally, the contrasting DNA extraction efficiency of both protocols resulted in notable alterations in significant phenotypic associations with microbial taxa relative abundances. In this case, the FSK protocol yielded a higher number of significant associations in both cohorts, with a low replication rate between protocols. In fact, none of the significant associations observed in samples from the LLD cohort extracted with this procedure were replicated with the APK method, while only 42% were replicated by both DNA extraction protocols in the 500FG cohort. To further evaluate the impact of integrative analyses that combine data generated with different methodological approaches on microbial–host associations, we compared the association results of alternative datasets integrating samples from both cohorts and DNA extraction methods by applying linear mixed models. When compared to the association profiles obtained from uniformly processed samples, the integrative analysis revealed a substantial change in the microbial–host association profiles. Methodological differences were found to alter between 25 and 50% of the associations between taxa relative abundances and host phenotypes. These findings highlight the potential for substantial disruption in association outcomes during integrative analyses, emphasizing the need for careful consideration of methodological harmonization in such studies.

This study has several limitations. Despite our efforts to limit technical variability that could complicate the assessment of differences directly caused by DNA extraction protocol, our samples are subject to technical heterogeneity at several levels. Firstly, while the isolation of LLD samples was done at the same time and place for both protocols, 500FG sample isolation with the APK and FSK methods was done in different laboratories and 4 years apart. Although only a minor effect size of storage time of extracted DNA has been previously reported^33,34^, inter-laboratory differences have been shown to impact microbial profiles, while having a limited effect on relative diversity levels^14^. Secondly, effectively assessing the impact of the bead-beating step would require a comparative analysis of both DNA extraction protocols with and without this additional step, so the design of the current study did not allow us to completely disentangle the effect of this step from that of the rest of the extraction procedure. Lastly, samples extracted with each protocol were sequenced in two different sequencing centers. While both centers applied the same sequencing technology, this difference could still represent another layer of technical variation, although previous studies have described sample sequencing to have a smaller impact than DNA extraction method^15^. Despite this, the inclusion of a mock community analysis with several technical replicates allowed us to exclusively assess the biases introduced by each DNA isolation method, limiting external sources of technical variability.

Notwithstanding these limitations, our study expands upon previous findings that the DNA extraction procedure used has a large impact on the recovered gut microbial diversity and structure. Importantly, while we compared only two DNA extraction protocols, our study allows us to draw relevant conclusions about the inability of the thermal lysis step to efficiently lyse gram-positive bacteria and the positive impact of bead-beating on the accurate recovery of bacterial diversity and composition, which is independent of the DNA extraction method used. To our knowledge, our analysis is the largest study to assess the impact of DNA extraction method and fecal sample processing on the recovered gut microbiome profiles, and our considerable sample size overcomes the statistical power limitations of earlier comparative analyses. This, in combination with the use of shotgun metagenomics, provides evidence of disruption of the species-level microbial profile by alternative DNA extraction methods. Although we only tested the differences in stool samples, it is likely that sufficient detection power will make it possible to unravel a similar effect in other microbiome samples, including those with lower biomass. In addition, we have demonstrated how the technical variability effect translates into the phenotype association analysis. This pinpoints the influence of DNA extraction methodology on biological conclusions and highlights the crucial role of methodological harmonization in microbiome research to ensure the validity and reliability of association findings. These results may help explain the considerable heterogeneity and low replication rate found in microbiome studies to date, an issue that has greatly hampered clinical translation of microbiome research findings.

## Conclusions

Altogether, our results highlight the need to account for the DNA extraction protocol used as a confounding factor in microbiome analyses. This is especially critical for multicenter studies and meta-analyses of data from multiple cohorts, an increasing focus of the microbiome research field.

### Supplementary Information

Supplementary Information 1.

Supplementary Tables.

## Data Availability

The datasets supporting the conclusions of this article are available in A) LLD: the European Genome-Phenome Archive (EGA, https://ega-archive.org) via accession number EGAC00001000457, https://ega-archive.org/dacs/EGAC00001000457 and B) 500FG: Sequence Read Archive (SRA, https://www.ncbi.nlm.nih.gov/sra) via accession numbers PRJNA319574, https://www.ncbi.nlm.nih.gov/bioproject/PRJNA319574 (APK protocol) and PRJNA942468 https://www.ncbi.nlm.nih.gov/bioproject/942468 (FSK protocol). Due to informed consent regulations, phenotypic data of the Lifelines-DEEP cohort are available upon request to Lifelines (https://www.lifelines.nl/researcher). Datasets can be made available under a data transfer agreement by contacting https://data.lifelines.nl and filling in the following form: https://docs.google.com/forms/d/e/1FAIpQLSccpKhH271fawj2E-6M8vyjdx11DPeOIZhhRLtg_zmOVFDotw/viewform. The data usage access is subject to local rules and regulations.

## Abbreviations

PCoA: Principal Coordinates Analysis
PERMANOVA: Permutational multivariate analysis of variance
CLR: Centered log-ratio
FDR: False discovery rate
BMI: Body mass index
IQR: Interquartile range
