# Supplementary material for: Choice of DNA extraction method affects stool microbiome recovery and subsequent phenotypic association analyses
Source: Sci Rep. 2024 Feb 16;14:3911. doi: 10.1038/s41598-024-54353-w (PMC10873414; doi:10.1038/s41598-024-54353-w)
Supplement: Supplementary file 1 — Supplementary Information 1. [file 41598_2024_54353_MOESM1_ESM.docx]

**Choice of DNA extraction method affects stool microbiome recovery and subsequent phenotypic association analyses**

Asier Fernández-Pato^1*^, Trishla Sinha^1^, Ranko Gacesa^1,2^, Sergio Andreu-Sánchez^1,3^, Milla F. Brandao Gois^1^, Jody Gelderloos-Arends^1^, Dianne B.H. Jansen^2^, Marloes Kruk^1^, Martin Jaeger^4^, Leo A.B. Joosten^4,5^, Mihai G. Netea^4,6^, Rinse K. Weersma^2^, Cisca Wijmenga^1^, Hermie J.M. Harmsen^7^, Jingyuan Fu^1,3^, Alexandra Zhernakova^1^, Alexander Kurilshikov^1^

**Current affiliations**

^1^ Department of Genetics, University of Groningen, University Medical Center Groningen, Groningen 9713GZ, the Netherlands

^2^ Department of Gastroenterology and Hepatology, University Medical Center Groningen, Groningen 9713GZ, the Netherlands

^3^ Department of Pediatrics, University of Groningen, University Medical Center Groningen, Groningen 9713GZ, the Netherlands

^4^ Department of Internal Medicine and Radboud Center for Infectious Diseases, Radboud University Medical Center, Nijmegen, the Netherlands

^5^ Department of Medical Genetics, Iuliu Hatieganu University of Medicine and Pharmacy, Cluj-Napoca, Romania

^6^ Department of Genomics and Immunoregulation, Life and Medical Sciences Institute, University of Bonn, Bonn, Germany

^7^ Medical Microbiology and Infection Prevention, University of Groningen, University Medical Center Groningen, Groningen 9713GZ, the Netherlands

*** Correspondence:**Alexander Kurilshikov
alexa.kur@gmail.com

**Supplementary Methods. Detailed step-by-step description of DNA extraction protocols.** Deviations from manufacturer’s instructions are specified (*).

1. **AllPrep DNA/RNA Mini Kit**
2. *Start with 100 mg of stool. If the sample is not in any storage buffer proceed to step 2. Otherwise, vortex the sample for 10 sec, centrifuge the tube for 10 min at maximum speed and discard supernatant.
3. *Add 110 μl of TE buffer containing lysozyme and proteinase K to the stool pellet and vortex for 10 sec.
4. *Incubate at room temperature (15–25°C) for 10 min. Incubate on a shaker or vortex for 10 sec at least every 2 min.
5. *Add 1200 μl of RLT Plus w/ β-ME to sample tube and vortex for 10 sec.
6. *Transfer sample into a 2 ml sterile bead-beating tube with 1 ml of 0.1-mm glass beads. Fill the remaining volume of the tube with RLT Plus w/ β-ME. Place the tube in the bead beater for 3 min.
7. *Centrifuge the tube for 5 min at max speed and transfer the supernatant into a sterile 1.5 ml tube.
8. *Centrifuge for 2 min at max speed to remove any additional debris.
9. Add the supernatant into a QIAshredder spin column placed in a 2 ml collection tube, and centrifuge for 2 min at max speed.
10. Transfer the homogenized lysate to the AllPrep DNA spin column placed in a 2 ml collection tube and centrifuge for 30 sec at ≥8,000 x g (≥10,000 rpm).
11. Place the AllPrep DNA spin column in a new 2 ml collection tube (supplied), and store at room temperature (15–25°C) or at 4°C.
12. Add 500 μl Buffer AW1 to the AllPrep DNA spin column. Centrifuge for 15 sec at ≥8,000 x g to wash the spin column membrane. Discard the flow-through.
13. Add 500 μl Buffer AW2 to the AllPrep DNA spin column. Centrifuge for 2 min at max speed to wash the spin column membrane.
14. Place the AllPrep DNA spin column in a new 1.5 ml collection tube. Add 50 μl Buffer 1x low TE or EB to the spin column membrane. Incubate at room temperature (15–25°C) for 1 min and centrifuge for 1 min at ≥8,000 x g (≥10,000 rpm) to elute the DNA.
15. Repeat the previous step to elute further DNA. To prevent dilution of the first DNA eluate, use a new 1.5 ml collection tube to collect the second DNA eluate and combine them.
16. Store the DNA at -20°C.
17. **QIAamp Fast DNA Stool Mini Kit**
18. Start with 180–220 mg of stool. Transfer the sample to a 2 ml microcentrifuge tube (Safe-Lock tube, Eppendorf).
19. Prepare the QIAcube machine for step 8, including the following components: Buffer AL, Buffer AW1, Buffer AW2, Proteinase K and Ethanol.
20. Add 1 ml InhibitEX Buffer to the sample. Vortex continuously for at least 1 min or until the stool sample is thoroughly homogenized.
21. *Heat the suspension for 10 min at 95°C, while shaking at 1000 rpm. Then, vortex each sample for 15 sec.
22. Centrifuge samples at max speed for 1 min to pellet stool particles at 20,000 x g (approximately 14,000 rpm).
23. Transfer 200 μl of the supernatant to a 2 ml microcentrifuge tube (Screw Cap tube).
24. Load the samples to the QIAcube robot, program on the Qiacube for QIAamp® Fast DNA Stool Mini Kit and follow the instructions.
25. Once the QIAcube is finished, store the DNA at -20°C.

**Supplementary Figures**


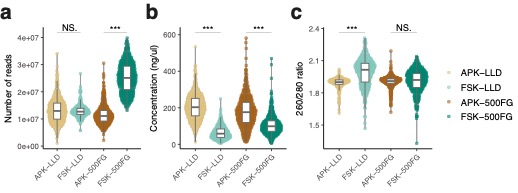


**Supplementary Figure 1. Read depth, DNA concentration and purity analysis.** Comparison of (A) the number of reads after contaminant removal, (B) DNA concentration (ng/μl) and (C) DNA purity measured as 260/280 absorbance ratio between samples extracted with the APK (brown) and FSK (green) protocols from each cohort (LLD: light color, 500FG: dark color). Each dot represents one sample. Boxplots illustrate the median (middle horizontal line), IQR (boundaries of the boxes) and values within 1.5 times the IQR (whiskers) (*** p-value < 0.001, rank-based Wilcoxon test).

**
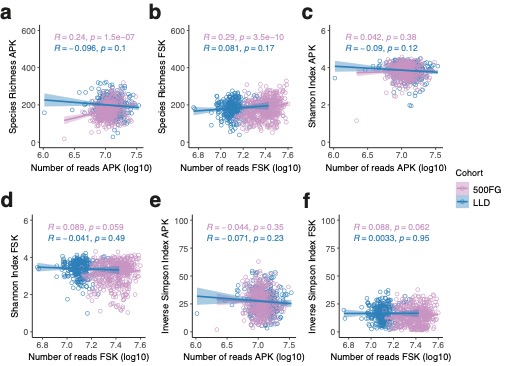
**

**Supplementary Figure 2.** **Correlation analysis between alpha diversity and read depth**. Correlation plots between species richness (A–B), Shannon (C–D) and Inverse Simpson (E–F) diversity indices and the number of reads after contaminant removal (log10 scale). Samples from each cohort are displayed (LLD: blue, 500FG: violet). Each circle is one sample. Coefficients and p-values from the Pearson correlation are shown.


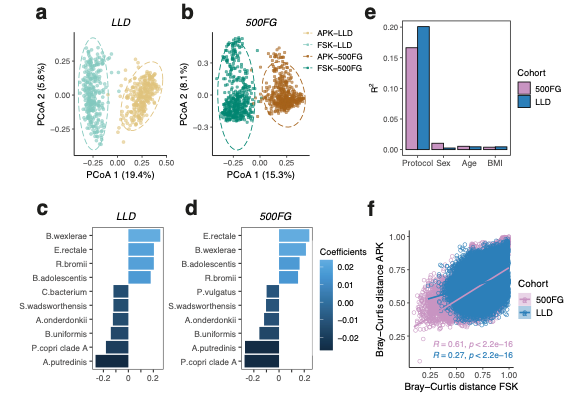


**Supplementary Figure 3.** **Beta diversity analysis based on Bray-Curtis dissimilarity**. PCoA plot for (A) LLD and (B) 500FG samples extracted with each DNA isolation method (APK: brown, FSK: green). Each datapoint represents the microbial community composition of one sample. Ellipses illustrate the standard deviation of samples belonging to each group. (C–D) Model coefficients estimated by PERMANOVA analysis for the species that exhibited the largest differences between samples extracted with the APK and FSK protocols in each cohort. The top 10 species are shown, ordered according to the coefficient value (continuous color scale: positive (increased in APK) in light blue and negative (increased in FSK) in dark blue). (E) Individual effect sizes, estimated by PERMANOVA, of DNA extraction protocol, sex, BMI and age on microbiome community variation (LLD: blue, 500FG: violet). (F) Correlation plot of Bray-Curtis distances between differentially extracted samples from each cohort (LLD: blue, 500FG: violet). Each data point represents one sample. Coefficients and p-values from the Mantel test are shown.

**Supplementary Figure 4.** **Core taxa analysis**. Each sub-cohort is analyzed separately: (A) APK-LLD, (B) FSK-LLD, (C) APK-500FG and (D) FSK-500FG. Heatmaps show the variation in taxa prevalence (continuous color scale, 100: red, 0: blue) according to relative abundance thresholds (y-axis). Only microbial species with a prevalence >90% are shown. Line plots show the alteration in the number of species present (y-axis) with changes in relative abundance (x-axis) for 50% and 90% prevalence.

**Supplementary Figure 5.** **Mock community analysis.** Comparison of **(A)** DNA concentration (ng/μl) and **(B)** Shannon diversity values between mock community samples extracted with the APK (brown) and FSK (green) protocols. A red horizontal line is shown indicating the theoretical Shannon diversity of the mock community. Each dot represents one sample. The sample extracted with the FSK protocol including a bead-beating step is highlighted (FSK + BB). Boxplots illustrate the median (middle horizontal line), IQR (boundaries of the boxes) and values within 1.5 times the IQR (whiskers). **(C)** PCoA plots based on Bray-Curtis (left) and Aitchison (right) distances of samples extracted with each DNA isolation method. Each data point represents the microbial community composition of one sample. The theoretical composition is indicated with a red dot. **(D)** Density plots showing the differences in Firmicutes and Proteobacteria relative abundances between samples extracted with the APK and FSK protocols. **(E)** Bar plot with species-level relative abundances of APK and FSK samples, including the theoretical composition.


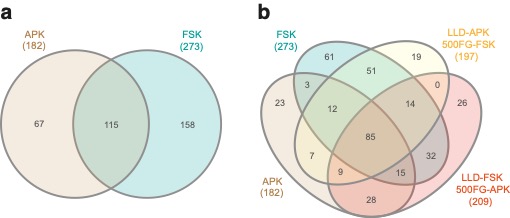


**Supplementary Figure 6.** **Association between host phenotypes and microbial relative abundances.** Venn diagrams displaying the comparison of significant associations between CLR-transformed microbial relative abundances and host phenotypes in **(A)** all samples extracted with the APK DNA extraction protocol in comparison to all samples extracted with the FSK protocol and (**B**) for combinations of samples from different cohorts (LLD and 500FG) extracted with the same or alternative DNA extraction protocols.
